# Spatial Choroidal Analysis under Mid-Phase Hyperfluorescent Plaques Demonstrates Unique Signatures in Central Serous Chorioretinopathy: Macula Society International Central Serous Chorioretinopathy Research Network Report Number 20

**DOI:** 10.1016/j.xops.2026.101404

**Published:** 2026-09-06

**Authors:** Giulia Gregori, Niroj Kumar Sahoo, Nasiq Hasan, Atharva Ajay Vichare, Shreyaa Rohindra Lall, Sharat Chandra Vupparaboina, Adarsh Gadari, Oscar Matteo Gagliardi, Marco Lupidi, Cesare Mariotti, Stanley Saju, Jessica Cao, Charles C. Wykoff, Kiran Kumar Vupparaboina, Sandeep Chandra Bollepalli, Lihteh Wu, Jay Chhablani, MICRoN Study Group

**Affiliations:** 1 Eye Clinic, Department of Experimental and Clinical Medicine, Polytechnic University of Marche, Ancona, Italy; 2 UPMC Eye Centre, University of Pittsburgh, Pittsburgh, Pennsylvania; 3 Retina Consultants of Texas, Houston, Texas; 4 Asociados de Macula Vitreo y Retina de Costa Rica, San José, Costa Rica

**Keywords:** CSC, MPHP, ER.

## Abstract

**Purpose:**

To evaluate the spatial relationship between mid-phase hyperfluorescent plaques (MPHP) and choroidal vascularity in central serous chorioretinopathy (CSCR) using choroidal vascularity index (CVI) heat maps and enrichment ratio (ER) analysis.

**Design:**

Retrospective multicenter study.

**Subjects:**

Forty eyes from 40 patients with CSCR presenting MPHP on indocyanine green angiography (ICGA) at both baseline and follow-up were included.

**Methods:**

Choroidal vascularity index was calculated from spectral-domain OCT scans using an automated algorithm generating en face CVI heat maps. Mid-phase hyperfluorescent plaques areas were manually segmented after overlaying with ICGA images. Spatial association between MPHP and high-CVI regions was assessed using ER analysis at absolute CVI thresholds of 0.75 and 0.90. Eyes were further stratified according to MPHP area dynamics (increased vs. decreased) at follow-up.

**Main Outcome Measures:**

Mean CVI inside versus outside the MPHP area; ER values at CVI thresholds of 0.75 and 0.90; intralesional CVI distribution; ER values by MPHP area dynamics subgroup.

**Results:**

Mean CVI was significantly higher within the MPHP area (0.72 ± 0.09) compared with the surrounding choroid (0.70 ± 0.09; paired Wilcoxon *P* < 0.01; mean difference, 0.022; 95% confidence interval, 0.010–0.033; Cohen d = 0.57). Enrichment ratio analysis demonstrated preferential localization of MPHP within high-CVI regions at the CVI threshold of 0.90 (ER > 0.90 = 1.67). Qualitative assessment revealed nonuniform intralesional CVI distribution in 70% of eyes. In subgroup analysis, eyes with increasing MPHP area showed a directional trend toward greater enrichment within regions of highest CVI values (ER > 0.90: median 1.43 vs. 0.84; *t* test *P* = 0.04, Mann‑Whitney *P* = 0.260), an exploratory finding.

**Conclusions:**

Mid-phase hyperfluorescent plaques in CSCR are spatially associated with regions of increased choroidal vascularity, supporting a spatially driven model of disease. These hypothesis-generating findings highlight the potential value of CVI-based spatial mapping for improving disease characterization and, pending prospective validation, therapeutic targeting.

**Financial Disclosure(s):**

Proprietary or commercial disclosure may be found in the Footnotes and Disclosures at the end of this article.

Central serous chorioretinopathy (CSCR) is a pachychoroid spectrum disorder characterized by serous neurosensory retinal detachment associated with alterations in choroidal circulation and permeability, occurring more commonly in middle-aged men.^1^^,^^2^ Increasing evidence suggests that the primary pathophysiological driver of CSCR is choroidal vascular dysfunction, including venous congestion, choroidal hyperpermeability, and structural remodeling of the choroidal vasculature.^3^

Although new developments in OCT have improved imaging capabilities, indocyanine green angiography (ICGA) remains the gold standard imaging method to identify the dynamic choroidal vascular changes occurring in CSCR.^4^ Early-phase ICGA demonstrates delayed choroidal filling together with dilation of choroidal veins. During the mid-phase, geographic areas of hyperfluorescence, also known as mid-phase hyperfluorescent plaques (MPHP), become evident; they increase in intensity until 7 to 10 minutes and do not show expansion during the course of the angiogram.^5^^,^^6^ They have well-defined borders and disappear at the very late phase of the angiogram (>20 minutes). Such regions of hyperfluorescence are reported in 59.4% of CSCR cases by Bousquet et al,^5^^,^^7^ with a higher frequency among those with chronic disease features, irregular pigment epithelial detachment, and foveal pachyvessels. This figure specifically refers to well-defined MPHP and should be distinguished from the broader phenomenon of diffuse ICGA choroidal hyperpermeability, which has been reported at higher frequencies in earlier literature.^8^

In a previous study, our group noted that these plaques have been attributed to choroidal vessels permeability and could gradually evolve into focal retinal pigment epithelium (RPE) alterations, potentially reflecting localized alterations at the RPE–choroid interface related to abnormal dye accumulation within dysfunctional RPE.^9^^,^^10^ These observations highlight the importance of not only identifying MPHP as angiographic markers of choroidal dysfunction but also understanding the underlying vascular environment in which they develop.

In parallel with angiographic studies, more recently, the choroidal vascularity index (CVI), defined as the ratio between the luminal area and the total choroidal cross-sectional area, has emerged as a quantitative and reproducible parameter for assessing choroidal vascular status in pachychoroid diseases.^11^^,^^12^ Previous studies have reported increased CVI values in eyes with CSCR compared with fellow or healthy eyes, supporting the concept of a vascular-dominant choroid in this condition.^13^^,^^14^ However, absolute CVI values derived from global measurements may have limited ability to reflect the spatial heterogeneity of choroidal vascular remodeling.^15^ In contrast, the spatial distribution of choroidal vascularity may play a critical role in determining retinal alterations and angiographic patterns. Recently, spatial analytical approaches have been proposed to evaluate the relationship between pathological features and the distribution of choroidal vascularity. Enrichment ratio (ER) analysis allows an assessment of whether a given structure preferentially localizes within areas of higher or lower CVI values. Such spatial metrics may provide additional insights beyond conventional mean CVI measurements by capturing regional vascular heterogeneity.

The aim of the present study was, therefore, to investigate the spatial relationship between MPHP and choroidal vascularity in CSCR using CVI heat maps and ER analysis. We analyzed the overall MPHP area and within it, intralesional variation in CVI to assess whether spatial gradients in choroidal vascularity exist within MPHP. Finally, we explored whether differences in the spatial distribution of MPHP were associated with changes in plaque area over time.

## Methods

This was designed as a retrospective, multicenter study that was part of the Macula Society CSCR project‑Macula Society International CSCR Research Network. The study adhered to the tenets of the Declaration of Helsinki and was approved by the local institutional review boards or ethics committees of each participating center. Data sharing agreements among collaborators and the procedures for demographic, clinical, and imaging data collection and analysis have been detailed in prior publications of the project. Inclusion criteria consisted of (1) patients aged >18 years, (2) a confirmed diagnosis of CSCR, and (3) presence of baseline and follow-up MPHP at ICGA. Exclusion criteria were (1) eyes with inadequate records or (2) coexisting retinal diseases, such as retinal vasculopathies, high myopia, or glaucomatous optic neuropathy.

Baseline demographic and systemic information was obtained. Best-recorded visual acuity was documented and expressed in logarithm of the minimum angle of resolution. Simple CSCR referred to cases where the disease was unifocal and confined to ≤2 disc area of RPE alteration, whereas complex CSCR included cases with multifocal changes or lesions extending beyond 2 disc area.^16^ Central serous chorioretinopathy was categorized as either acute or chronic based on the duration of serous retinal detachment; eyes with serous retinal detachment persisting for >3 to 6 months are described as having chronic CSCR.^17^ The groups were further stratified into increased MPHP area (group 1) and decreased MPHP area (group 2), compared with the MPHP area at baseline, for subgroup analysis. The patients were followed clinically for a median of 29.3 months (interquartile range [IQR], 11.7‑40.5 months). For the subgroup analysis of MPHP dynamics, the analysis was restricted to eyes under observation only, minimizing treatment-related confounding. Of the 40 eyes in the overall cohort, 2 eyes received treatment between the baseline and follow-up imaging assessments and were therefore excluded from the subgroup analysis of MPHP area dynamics. Imaging data were also collected. All images were meticulously analyzed, and the MPHP area was measured by an experienced retina specialist (N.K.S.); 20 images (50% of the cohort) were independently reviewed and validated by another masked grader (G.G.) to ensure accuracy and consistency. In case of disagreements, the measurements and segmentations were adjudicated by the senior author (J.C.).

### Image Processing

Spectral-domain OCT imaging was acquired using the Spectralis High Resolution Angiographhy + OCT system (Heidelberg Engineering) after pharmacologic pupil dilation. Only scans with a signal strength of ≥7/10 were included to ensure reliable measurements. A standardized 6 × 6 mm volume scan protocol (49 B-scans) was performed for all eyes. Choroidal vascularity index was quantified using a previously validated automated algorithm that incorporates shadow compensation, noise reduction, delineation of inner and outer choroidal boundaries, and segmentation of the choroid from the overlying retinal structures.^18^ Each B-scan was divided into multiple A-scans, which were binarized in MATLAB (Matrix Laboratory) to distinguish hyper-reflective stromal areas from hyporeflective luminal regions. Choroidal vascularity index was calculated as the ratio of luminal area to total choroidal area, allowing the generation of an en face matrix representing pixel-wise CVI distribution.^19^ For localized CVI analysis, a custom CVI Calculation Tool was used. For each eye, the corresponding infrared image delineating the scan area, along with the MATLAB (.mat) file containing the raw CVI matrix and choroidal volume data, was imported into the software. Manual superimposition (of the en face image) was performed to define the overall MPHP area after alignment with the mid-ICGA phases. Superimposition was performed by 2 trained graders (G.G. and N.K.S.), and all delineations were independently validated. Registration accuracy was assessed qualitatively by visual confirmation of structural overlap at major anatomical landmarks. The spatial distribution of CVI within plaques was qualitatively assessed to explore potential intralesional heterogeneity. The rationale for defining this heterogeneity was based on the visual inspection of CVI heat maps, which consistently revealed a focal subregion of elevated vascularity near the center of MPHP. The area outside the MPHP polygon within the entire scan frame served as the reference zone for CVI comparisons.

Pixels were, therefore, categorized into 2 zones: inside the overall MPHP area and outside the MPHP polygon (i.e., the surrounding choroid). For each zone, the tool computed mean CVI, standard deviation, and range.

To quantify the distribution of choroidal vascularity within each MPHP area, threshold analysis was performed to determine the proportion of pixels exceeding absolute CVI cutoffs of 0.75 and 0.90. These thresholds were selected to capture 2 levels of vascular enrichment: a CVI value >0.75 representing areas of moderately increased choroidal vascularity and a CVI value >0.90 representing regions of the highest vascular density, corresponding to the most dilated and vascular-dominant portions of the choroid. Pixels exceeding these thresholds were defined as belonging to the high-CVI zone for the respective cutoff level; all remaining pixels constituted the low-CVI zone. The ER classification thresholds of >1.2 (enriched), 0.8 to 1.2 (neutral), and <0.8 (depleted) represent a ±20% deviation from the null expectation of ER = 1.0 (no spatial preference) and have been applied in analogous spatial enrichment in retinal imaging research. In particular, this absolute threshold approach was adopted based on cutoffs previously defined in our prior work, allowing biologically interpretable cutoffs to be applied consistently across eyes.^20^

### ER Analysis

To evaluate the preferential spatial localization of the MPHP area, ER were calculated at both CVI thresholds (>0.75 and >0.90). The ER was defined as:

Enrichment ratio = P (high CVI | zone of interest)/

P (high CVI |overall frame)

where P (high CVI | zone of interest) is the proportion of pixels exceeding the CVI threshold within the zone of interest (overall MPHP area) and P (high CVI | overall frame) is the baseline proportion of high-CVI pixels across the entire scan frame, serving as the expected frequency under the null hypothesis of no spatial preference. In the primary analysis, the reference denominator for the ER calculation was the entire scan frame, inclusive of the MPHP polygon itself. To assess whether this choice influenced the results, we performed a sensitivity analysis in which the denominator was restricted to the choroidal area outside the MPHP polygon only, thereby excluding the MPHP region from the reference frequency of high-CVI pixels.

Enrichment ratio values >1.2 indicated preferential localization within high-CVI regions, values <0.8 indicated preference for low-CVI areas, and values between 0.8 and 1.2 indicated no spatial preference. The CVI >0.90 threshold was selected as the primary threshold for the main analysis, as it identifies pixels with the highest degree of choroidal vascularity and therefore most directly captures focal vascular dominance. The CVI threshold value >0.75 was retained for secondary analysis to assess the consistency of spatial enrichment across a broader range of vascular activity.

### Statistical Analysis

All analyses were conducted using R Studio (version 2025.09.1+40), R Foundation for Statistical Computing. Continuous variables were summarized as mean ± standard deviation or median (IQR), whereas categorical variables were expressed as counts and percentages. The normality of data distribution was examined using the Shapiro-Wilk test. Comparisons between groups were made using the independent-samples *t* test or Mann‑Whitney U test for continuous variables and the χ^2^ or Fisher exact test for categorical data.

As the comparison of CVI between the MPHP area and the surrounding choroid, as well as the comparison of CVI standard deviation inside versus outside the MPHP, involved 2 measurements derived from the same eye, these specific comparisons were performed using a paired *t* test or Wilcoxon signed-rank test, as appropriate based on the normality of the paired differences.

Given the exploratory nature of this study, no formal correction for multiple comparisons was applied. Results are, therefore, considered hypothesis-generating rather than confirmatory; *P* values near 0.05 should be interpreted with caution in the context of multiple comparisons.

The interclass coefficient was calculated between the masked graders (N.K.S. and G.G.) for measurements in the MPHP area and CVI measurements. A *P* value of <0.05 was considered statistically significant.

## Results

### Demographic Data

This study analyzed 40 eyes from 40 patients with CSCR. The mean age of the patients was 51.3 ± 10.6 years. Among the subjects, 4 (10%) were females. The mean best-recorded visual acuity was 0.23 ± 0.26 logarithm of the minimum angle of resolution (approximate Snellen equivalent 20/32). Only 1 of the 40 eyes (2.5%) had a documented history of prior treatment before the baseline visit; the remaining 39 eyes were treatment-naïve at baseline. Baseline characteristics have been summarized in Table 1. The groups were further stratified into: increased MPHP area (group 1) and decreased MPHP area (group 2).

**Table 1:** Baseline Characteristics

Parameter	Overall (n = 40)	Group 1 (n = 13) Increased MPHP Area	Group 2 (n = 25) Decreased MPHP Area	*P* Value (1-2)
Age, years	51.3 ± 10.6	53.31 ± 7.59	50.44 ± 8.29	0.77
Female (%)	4 (10%)	2 (15%)	4 (16%)	0.43
Simple (%)	23 (57.5%)	7 (53.8%)	16 (64%)	0.53
Chronic (%)	38 (95%)	13 (100%)	23 (92.6%)	1
BRVA, logMAR	0.21 ± 0.2	0.33 ± 0.30	0.13 ± 0.11	**0.003**
Overall CVI	0.7 ± 0.09	0.72 ± 0.1	0.7 ± 0.09	0.55
CVI in overall MPHP	0.72 ± 0.09	0.73 ± 0.1	0.72 ± 0.08	0.64
CVI outside the polygon	0.7 ± 0.09	0.71 ± 0.1	0.70 ± 0.09	0.57
ER >0.75 MPHP overall	1.06 ± 0.38	1.12 ± 0.38	0.95 ± 0.39	0.88
ER >0.90 MPHP overall	1.67 ± 2	3.03 ± 5.03	1.02 ± 0.58	*t* test: **0.04**; Mann-Whitney (MW) test: 0.260∗
CMT, micron	322.09 ± 105.84	339 ± 107.43	303.46 ± 90.68	0.31
NSD, micron	134.13 ± 137	167.22 ± 157.22	64.92 ± 57.47	**0.03**
SFCT, micron	362.82 ± 106.31	376.11 ± 93.47	354.92 ± 116.08	0.53
Baseline MPHP area, mm^2^	10.09 ± 7.78	11.99 ± 9.19	9.25 ± 7.33	0.31

BRVA = best-recorded visual acuity; CMT = central macular thickness; CVI = choroidal vascularity index; ER = enrichment ratio; logMAR = logarithm of the minimum angle of resolution; MPHP = mid-phase hyperfluorescent plaques; NSD = neurosensory retinal detachment; SFCT = subfoveal choroidal thickness.

Bold values indicate statistical significance (*P* < 0.05).

∗ Independent *t* test *P* = 0.04; Mann‑Whitney U *P* = 0.260.

### Comparison of Choroidal Vascularity in the MPHP Area and in the Outside Region

Comparison of CVI values between regions revealed significant differences across MPHP and the surrounding choroid. The mean CVI within the MPHP area (0.72 ± 0.09) was significantly higher than the outside region (0.70 ± 0.09). As both measurements were derived from the same eye, this comparison was performed using a paired Wilcoxon signed-rank test (*P* < 0.01). The mean difference was 0.022 CVI units (95% confidence interval: 0.010–0.033), corresponding to a medium effect size (Cohen d = 0.57). Within the entire MPHP region, CVI remained higher in eyes with increasing MPHP area (0.74 ± 0.10) than in those with decreasing MPHP area (0.71 ± 0.08), although this difference did not reach statistical significance (*P* = 0.64). In contrast, CVI measured outside the MPHP polygon showed nearly identical values between groups (0.71 ± 0.10 in group 1 vs. 0.70 ± 0.09 in group 2, *P* = 0.57, Fig 1).

**Figure 1:**
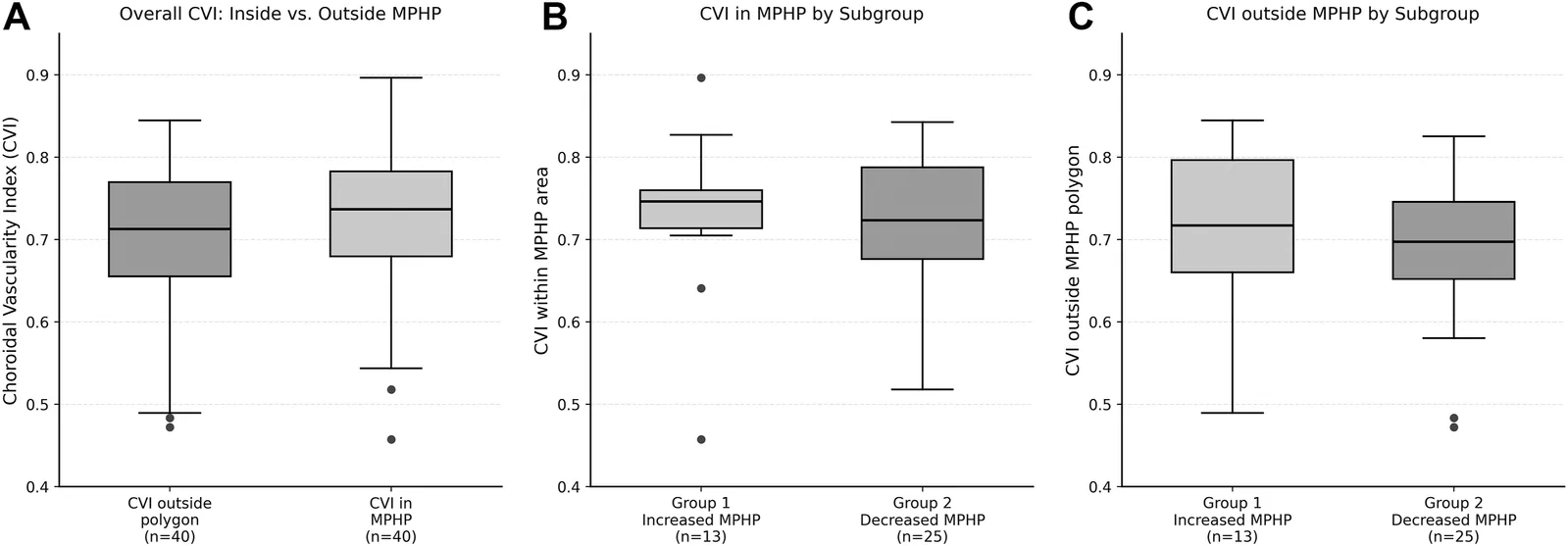
Box plots showing distribution of CVI values in **(A)** the overall MPHP area and outside the MPHP polygon in the entire cohort, **(B)** within the MPHP area in group 1 (increased MPHP area) and group 2 (decreased MPHP area), and **(C)** outside the MPHP polygon in group 1 (increased MPHP area) and group 2 (decreased MPHP area). CVI = choroidal vascularity index; MPHP = mid-phase hyperfluorescent plaques.

### ER Analysis: Overall Spatial Distribution of MPHP Area

Enrichment ratio analysis demonstrated the differential spatial distribution of MPHP across CVI thresholds. Using the CVI threshold of 0.75, the ER within the overall MPHP area was 1.06, indicating no clear spatial preference for high-CVI regions (neutral range, 0.8–1.2). However, at the more stringent CVI threshold of 0.90, ER increased to 1.67 in the overall MPHP area, indicating preferential localization within the highest CVI region. A sensitivity analysis using the outside-MPHP region as the reference denominator yielded comparable results to the primary analysis: ER >0.75 was 1.03 ± 0.45 and ER >0.90 was 1.66 ± 2.99 overall, closely matching the primary estimates (1.06 and 1.67, respectively). In subgroup comparisons, ER >0.90 using this alternative denominator was higher in group 1 (median, 1.44; IQR, 0.80–2.54) than in group 2 (median, 0.83; IQR, 0.60–1.33; *P* = 0.06), consistent in direction with the primary analysis. Moreover, the distribution of ER values also demonstrated variability across ER thresholds. Within the overall MPHP area, at the CVI threshold of 0.75, intermediate ER values (0.8–1.2) were most frequently observed (21 eyes; 52.5%), followed by ER >1.2 in 13 eyes (32.5%) and ER <0.8 in 6 eyes (15.0%). When applying the CVI threshold of 0.90, the distribution was more balanced, with ER >1.2 observed in 15 eyes (37.5%), intermediate values in 15 eyes (37.5%), and ER <0.8 in 10 eyes (25.0%) (Table 1, Fig 2).

**Figure 2:**
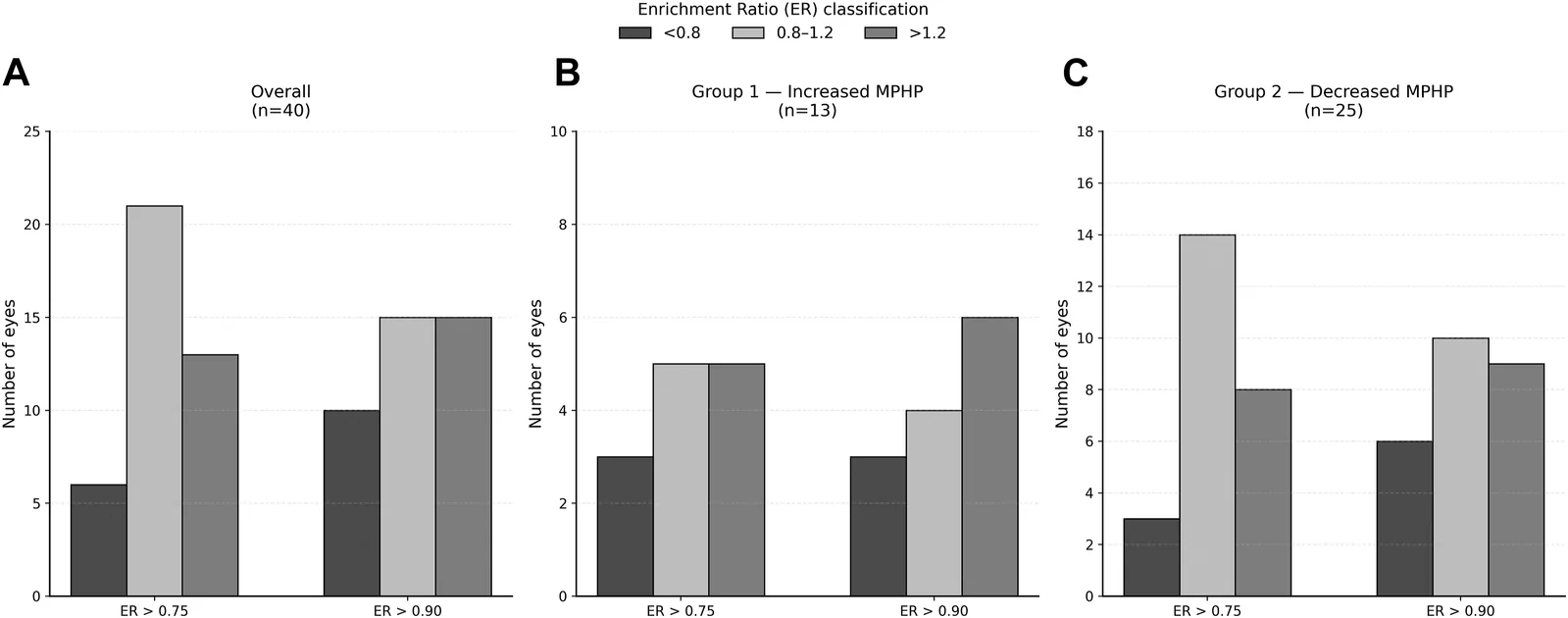
Bar charts showing the distribution of eyes across enrichment ratio (ER) categories (<0.8, 0.8–1.2, >1.2) at absolute CVI thresholds of 0.75 and 0.90, for **(A)** the overall cohort (n = 40), **(B)** group 1—increased MPHP area (n = 13), and **(C)** group 2—decreased MPHP area (n = 25). CVI = choroidal vascularity index; MPHP = mid-phase hyperfluorescent plaques.

### ER Analysis: Spatial Distribution of MPHP in Different Subgroups

In the subgroup analysis, when considering the CVI threshold of 0.75, the ER within the overall MPHP area was comparable between the 2 groups (1.12 ± 0.38 in eyes with increased MPHP area vs. 0.95 ± 0.39 in eyes with decreased MPHP area; *P* = 0.88). At the more stringent CVI threshold of 0.90, a different pattern emerged. The overall MPHP area showed numerically higher enrichment in eyes with increasing MPHP area compared with those with decreasing plaques, although this difference did not reach statistical significance (ER >0.90: median 1.43, IQR 0.75–2.75 vs. median 0.84, IQR 0.63–1.42; Mann‑Whitney U test, *P* = 0.260).

When stratified by MPHP area dynamics, ER distributions at CVI thresholds of 0.75 and 0.90 showed notable variability across groups. At the CVI threshold of 0.75, the increased MPHP group showed a nonuniform distribution pattern, with 5 eyes (38.5%) demonstrating ER >1.2, 5 eyes (38.5%) showing intermediate values (0.8–1.2), and 3 eyes (23.1%) exhibiting ER <0.8, whereas the decreased MPHP group was mainly characterized by intermediate ER values (14 eyes; 56%), followed by ER >1.2 in 8 eyes (32%), while ER <0.8 was observed in 3 eyes (12%). When applying the more stringent CVI threshold of 0.90, variability increased. In the increased MPHP group, ER >1.2 was observed in 6 eyes (46.2%), intermediate values in 4 eyes (30.8%), and ER <0.8 in 3 eyes (23.1%). In contrast, the decreased MPHP group demonstrated a broader distribution, with 10 eyes (40%) showing intermediate ER values, 9 eyes (36%) ER >1.2, and 6 eyes (24%) ER <0.8 (Table 1, Fig 2).

When comparing baseline structural parameters between groups, no significant differences were observed in most variables. The mean central macular thickness was 322.09 ± 105.84 μm in the overall cohort, 339.00 ± 107.43 μm in eyes with increased MPHP area, and 303.46 ± 90.68 μm in those with decreased MPHP area, without reaching statistical significance (*P* = 0.31).

In contrast, neurosensory detachment height differed significantly between groups (*P* = 0.03). Eyes with increased MPHP area demonstrated higher mean baseline neurosensory detachment height (167.22 ± 157.22 μm) compared to those with decreased area (64.92 ± 57.47 μm; *P* = 0.03). This difference may reflect greater disease activity or chronicity and should be interpreted cautiously.

No statistically significant differences were observed for subfoveal choroidal thickness, which measured 362.82 ± 106.31 μm overall, 376.11 ± 93.47 μm in the increased MPHP group, and 354.92 ± 116.08 μm in the decreased MPHP group (*P* = 0.53). Similarly, the baseline MPHP area did not significantly differ between groups (10.09 ± 7.78 mm^2^ overall; 11.99 ± 9.19 mm^2^ in the increased MPHP group; 9.25 ± 7.33 mm^2^ in the decreased MPHP group; *P* = 0.31), indicating that the subsequent change in plaque area was not primarily determined by baseline plaque size. The follow-up duration did not differ significantly between group 1 (median, 30.4 months; IQR, 9.5–31.5) and group 2 (median, 29.2 months; IQR, 14.9–41.1; Mann‑Whitney *P* = 0.740), indicating that differential observation time did not confound the subgroup comparison of MPHP dynamics.

Independent validation was performed by a second masked grader (G.G.) on 20 images. The interclass correlation coefficient between the measurements of MPHP area at baseline was 0.87 (95% confidence interval: 0.79–0.93; *P* < 0.01) and that during follow-up was 0.90 (95% confidence interval: 0.87–0.94; *P* < 0.01).

### Intralesional CVI Distribution

Visual inspection of CVI heat maps revealed heterogeneous patterns within individual plaques, with areas of relatively higher and lower CVI coexisting within the same MPHP. These gradients were observed as localized variations in CVI intensity across plaques in 28 of 40 cases (70%) (Fig 3). To further support this observation, we quantified the variability of CVI by calculating the standard deviation within and outside the MPHP regions. The standard deviation was numerically higher within the plaque (0.14 ± 0.04) compared to outside (0.13 ± 0.03), although this difference did not reach statistical significance and should be considered an exploratory observation only (*P* = 0.14) (Fig 3).

**Figure 3:**
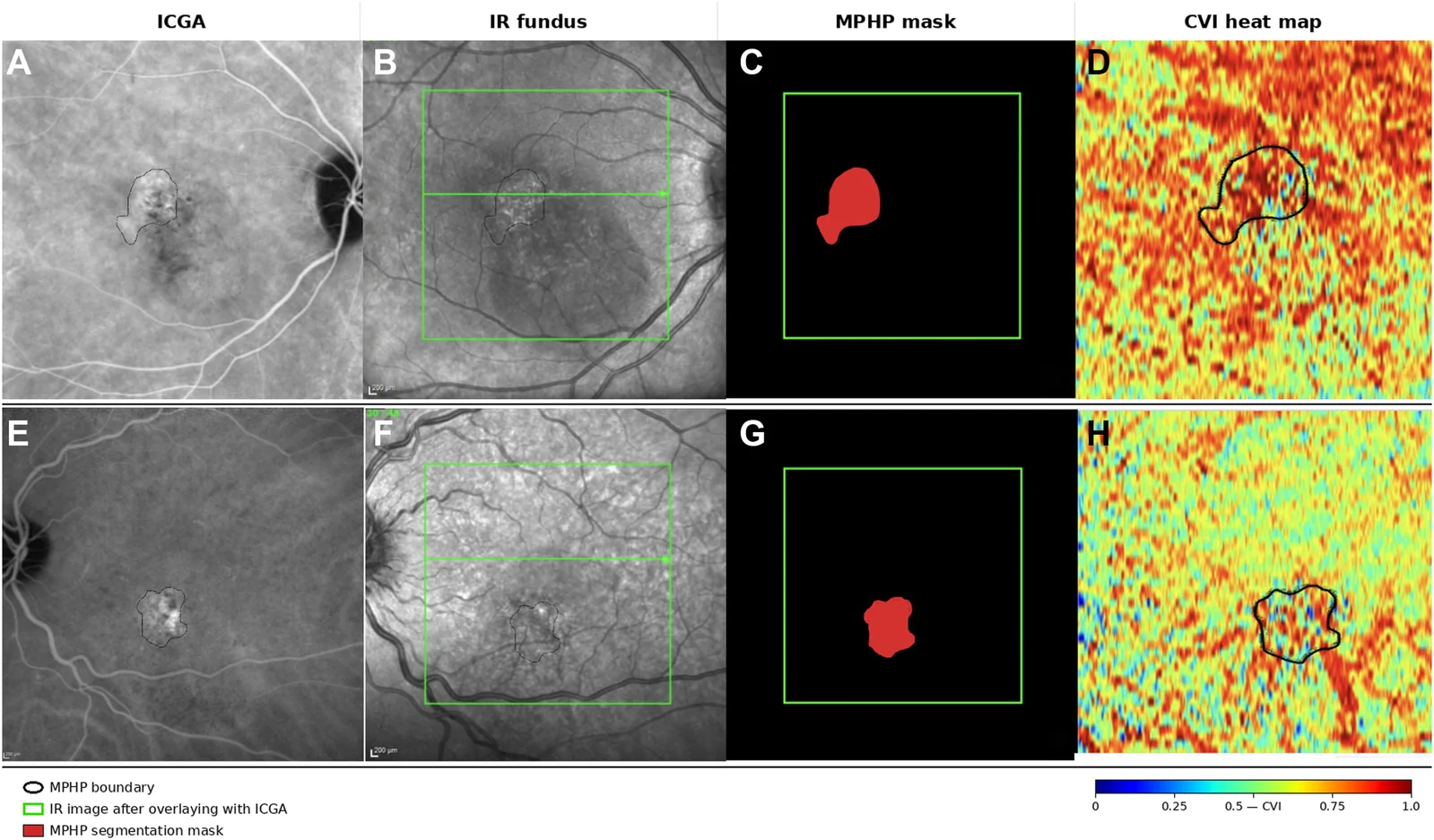
Example of a 50-year-old male CSCR patient with increased MPHP area at follow-up (top row, **A–D**). **(A)** Indocyanine green angiography mid-phase image with marked MPHP boundary. **(B)** Infrared fundus image after overlaying with ICGA, showing the OCT scan area (green rectangle) and MPHP boundary. **(C)** Binary MPHP segmentation mask. **(D)** En face CVI heat map with overlaid MPHP boundary. ER values: ER > 0.75 = 1.80, ER > 0.90 = 2.32; CVI inside MPHP = 0.80, CVI outside polygon = 0.78. The CVI heat map shows a nonuniform intralesional pattern with visible variations in signal intensity within the plaque. Example of a 47-year-old male CSCR patient with decreased MPHP area at follow-up (bottom row, **E–H**). **(E)** Indocyanine green angiography mid-phase image with marked MPHP boundary. **(F)** Infrared fundus image after overlaying with ICGA, showing the OCT scan area and MPHP boundary. **(G)** Binary MPHP segmentation mask. **(H)** En face CVI heat map with overlaid MPHP boundary. ER values: ER > 0.75 = 0.98, ER > 0.90 = 1.22; CVI inside MPHP = 0.70, CVI outside polygon = 0.64. The CVI heat map demonstrates intralesional heterogeneity with regional variations in signal intensity across the plaque. CSCR = central serous chorioretinopathy; CVI = choroidal vascularity index; ER = enrichment ratio; ICGA = indocyanine green angiography; IR = infrared; MPHP = mid-phase hyperfluorescent plaques.

## Discussion

The present study demonstrates that MPHP in CSCR shows a distinct spatial relationship with the underlying choroidal vascular architecture. Our study shows that MPHP preferentially localizes within regions characterized by higher CVI, supporting a nonrandom spatial association between angiographic abnormalities and the choroidal vascular environment. While statistically significant, the absolute CVI difference between MPHP and the surrounding choroid was numerically modest, consistent with a gradual vascular gradient rather than a discrete biological boundary. Importantly, rather than identifying a discrete subregion with a specific biological role, our analysis suggests that CVI within MPHP exhibits intralesional variability, indicating that these plaques may encompass heterogeneous choroidal vascular patterns.

This intralesional heterogeneity and the preference toward very high CVI zones suggest that MPHPs are organized around focal zones of vascular dominance rather than representing diffuse or randomly distributed areas of hyperpermeability. This supports the pachychoroid disease concept, in which localized choroidal venous congestion and dilation of large Haller vessels lead to focal remodeling of the choroidal circulation.^21^ Within this framework, areas of increased CVI likely reflect regions where the luminal component of the choroid predominates, potentially corresponding to zones of venous overload and increased hydrostatic pressure.^22^ Such hemodynamic alterations may compromise the integrity of the outer blood–retinal barrier and promote the development of ICGA hyperfluorescent plaques.^23^

The ER analysis further reinforces this interpretation. Mid-phase hyperfluorescent plaques demonstrated a tendency to localize within regions of highest absolute CVI values, particularly at more stringent thresholds, indicating that plaques are not merely associated with vascularized choroid but are specifically clustered in areas of extreme vascular enrichment. Notably, a directional trend was observed whereby eyes with increasing plaque area at follow-up showed higher median ER >90 enrichment compared with eyes demonstrating plaque reduction, although this difference did not reach statistical significance on nonparametric testing and should be regarded as exploratory. This pattern is consistent with the hypothesis that persistent focal vascular dominance may sustain MPHP expansion, but prospective studies with larger sample sizes are required to confirm this association. These observations support emerging models of CSCR pathophysiology emphasizing focal choroidal hemodynamic imbalance rather than generalized choroidal thickening.^24^^,^^25^ In pachychoroid disorders, dilated outer choroidal vessels are thought to compress the inner choroid, leading to structural remodeling, increased vascular permeability, and focal leakage phenomena.^26^ Mid-phase hyperfluorescent plaques may therefore represent the angiographic expression of these localized choroidal stress points, where vascular congestion and hyperpermeability converge.^5^ The focal zones of the highest CVI enrichment identified within MPHP may correspond to choroidal vascular structures recently described as synphlebia, interconnected Haller layer venous channels, potentially representing the structural OCT-based correlate of the vascular dominance identified in our spatial analysis.^27^

The analysis of intralesional CVI patterns in this study was exploratory and descriptive in nature. Although the difference between inside and outside the MPHP did not reach statistical significance, the observed trend toward greater intralesional variability supports the concept of heterogeneous vascular remodeling within MPHP. The lack of significance may be related to the limited sample size and intrinsic temporal heterogeneity of the underlying pathology, as eyes may have been captured at different stages of plaque evolution, thereby diluting measurable differences at a single time point. While regional differences in CVI were observed in 70% of eyes within MPHP, these findings should not be interpreted as evidence of distinct functional subcomponents within the plaque. Instead, they likely reflect underlying spatial variability in choroidal structure. A plausible explanation is that the heterogeneity in CVI distribution may be driven by localized alterations in the surrounding stromal environment, including stromal edema and expansion. This could lead to mechanical displacement and compression of adjacent vascular structures, with relative crowding of pachyvessels in some regions and apparent rarefaction in others. As a consequence, areas with higher CVI may correspond to zones of more prominent pachyvessel aggregation, whereas adjacent regions may reflect stromal swelling with secondary vascular redistribution.^11^

Identifying the specific patterns of CVI distribution (preference for very high CVI and lesion heterogeneity) within MPHP may have direct therapeutic implications. Photodynamic therapy is currently considered one of the most effective treatments for chronic or recurrent CSCR and is typically guided by areas of hyperfluorescence on ICGA.^28^ However, ICGA remains an invasive procedure with variable accessibility, inherent risks, and potential contraindications in some individuals. Using MPHP-specific CVI signatures may enhance the physiological precision of photodynamic therapy by focusing treatment on the zones with the greatest choroidal vascular overload, potentially offering a noninvasive imaging-based surrogate for ICGA in treatment planning and lesion guidance.^29^ Integrating ICGA plaque morphology with spatial CVI mapping could consequently provide a more refined strategy for treatment planning, enabling clinicians to identify the most pathologically relevant subregions within hyperfluorescent plaques. Beyond their potential value as candidate biomarkers for treatment guidance, these results also contribute to the understanding of disease progression, although prospective validation remains necessary before any clinical application can be considered. Eyes with increasing plaque area showed a directional trend toward greater enrichment within extreme CVI regions, suggesting that persistent vascular dominance may sustain or amplify the choroidal dysfunction underlying plaque expansion. Moreover, plaques with a more heterogeneous spatial distribution relative to CVI values may also reflect a more active and dynamic choroidal state characterized by stromal edema and transient vascular remodeling. Taken together, these findings highlight the importance of spatial vascular metrics in characterizing the choroidal microenvironment in CSCR.^30^ While global CVI measurements provide information about overall choroidal vascularity, they may fail to capture the localized vascular regions of higher CVI enrichment that seem to determine where angiographic abnormalities develop and how they evolve.^11^ By revealing that MPHP localizes in high-CVI zones that correspond to focal areas of maximal vascular enrichment, the present study provides further evidence that CSCR is fundamentally a disease of regional choroidal vascular imbalance within the pachychoroid spectrum, highlighting the importance of targeting the areas of the highest measured CVI values to improve treatment success and reduce recurrence.

Moreover, several limitations of this study should be acknowledged. Because inclusion required the presence of MPHP at both baseline and follow-up, this study necessarily excluded CSCR eyes without MPHP. As a result, our findings apply specifically to eyes with established MPHP and cannot be generalized to the broader CSCR population or used to predict where MPHP will develop. Second, the relatively small sample size limits statistical power and reduces generalizability. Third, the analyses were based on imaging obtained at a single time point to assess plaque dynamics. Although eyes were stratified according to subsequent changes in MPHP area, a fully longitudinal imaging approach would be necessary to determine whether spatial CVI enrichment precedes plaque expansion and could therefore serve as a predictive biomarker. In addition, the delineation of MPHP boundaries was based on manual segmentation of ICGA images, which may introduce some operator-dependent variability despite careful review. Residual misalignment could introduce spatial inaccuracy in the ER estimates, particularly in eyes with suboptimal landmark colocalization. Automated or semi-automated segmentation methods could improve reproducibility in future investigations. Moreover, the ER classification thresholds (>1.2, 0.8–1.2, <0.8) have been consistently applied by our group across spatial enrichment analyses; however, this scheme represents a methodological convention rather than a threshold independently validated against clinical or histopathological outcomes. Additionally, the definition of intralesional regions for CVI assessment was based on image-derived segmentation and should be interpreted as a methodological construct rather than an anatomically distinct entity. Finally, this study did not evaluate treatment response, and, therefore, the proposed implications regarding potential guidance for photodynamic therapy remain speculative. Prospective studies integrating spatial CVI mapping with treatment outcomes will be necessary to determine whether targeting high-CVI plaque cores can improve therapeutic efficacy.

## Conclusions

This study demonstrates that MPHP corresponds to focal regions of higher CVI enrichment, supporting a spatially driven model of CSCR and highlighting the potential value of CVI-based mapping for improving both disease understanding and treatment targeting. Future studies incorporating longitudinal, localized CVI changes and treatment effects may provide further insight into the role of the underlying choroidal vasculature.
