# Supplementary material for: Spatial Choroidal Analysis under Mid-Phase Hyperfluorescent Plaques Demonstrates Unique Signatures in Central Serous Chorioretinopathy: Macula Society International Central Serous Chorioretinopathy Research Network Report Number 20
Source: Ophthalmol Sci. 2026 Sep 6;6(11):101404. doi: 10.1016/j.xops.2026.101404 (PMC13657372; doi:10.1016/j.xops.2026.101404)
Supplement: Supplementary file 1 [file mmc1.docx]

**Supplemental Appendix**

*The Macula society for International CSCR Research Network (MICRoN) study group*

Nasiq Hasan, MD^1^; Arman Zarnegar, MD^1,2^; Jay Chhablani, MD^1,2^; Priyanka Gandhi, MD^1^; Carmen Antía Rodríguez-Fernández, MS^3^; Luis Arias Barquet, MD, PhD^3^; Jessica Cao, MD^4^; Stanley Saju, DO^4^; Charles C. Wykoff, MD, PhD^4^; Lisa Checchin, MD^5^; Maurizio Battaglia Parodi, MD^5^; Gabriele Piccoli, MSc, OD^5^; Lorenzo Pili, MD^5^; Peranut Chotcomwongse, MD^6^; Paisan Ruamviboonsuk, MD^6^; Lorenzo Ferro Desideri, MD, PhD^7^; Manjot K. Gill, MD, FASRS^7^; Felicia Hertkorn, MD^7^; Marion R. Munk, MD, PhD^7,8,9^; Halit Winter, MD^7^; Adrian T Fung, MBBS, MMED, FRANZCO^10^; Samer Khateb, MD, PhD^11^; Rahul N. Khurana, MD^12^; Jay Wang, MD^12^; Min Kim, MD, PhD, MBA, FASRS^13^; Yusuf Ashfaq, BS^14^; Zachary Kroeger, MD^14^; Timothy Lai, MD^15^; Luiz H. Lima, MD^16^; Stela Vujosevic, MD, PhD, FEBO^17,18^; Elizabeth Rossin, MD, PhD^19^; Priya Shah, BS^19^; Panisa Singhanetr, MD^19^; Lucia Sobrin, MD^19^; Ninan Jacob, MD^20^; Niroj Kumar Sahoo, MD^20^; Rufino Silva, MD^21,22^; Kent Small, MD^23,24^; Carol Villafuerte-Trisolini, MD^25^; Glenn Yiu, MD, PhD^25^; Sunir Garg, MD^26^; Bita Momenaei, MD^26^; Roselind Ni, MD^26^; Lihteh Wu, MD^27^; Michael Zhang, MD^28,29^; Giulia Gregori, MD^30^; Marco Lupidi, MD^30^; Elodie Bousquet, MD^31^; Andrea Coletto, MD^31^; Naoya Imanaga, MD^32^; Hideki Koizumi, MD^32^; Francisco Rodriguez, MD^33^

**Affiliations**

^1^University of Pittsburgh Medical Center, Pittsburgh, PA, USA

^2^University of Pittsburgh School of Medicine, Pittsburgh, PA, USA

^3^Department of Ophthalmology, Hospital Universitari de Bellvitge, Carrer de La Feixa Llarga, S/N, 08907, L'Hospitalet de Llobregat, Catalunya, Spain

^4^Retina Consultants of Texas, Retina Consultants of America, Blanton Eye Institute, Houston, TX, USA

^5^Ophthalmology Unit, IRCCS San Raffaele Scientific Institute, Milan, Italy

^6^Vitreoretina Unit, Department of Ophthalmology, Rajavithi Hospital, Rungsit University, Bangkok, Thailand

^7^Department of Ophthalmology, Northwestern University Feinberg School of Medicine, Chicago, IL, USA

^8^Inselspital, University Hospital Bern, Bern, Switzerland

^9^Gutblick, Pfäffikon, Switzerland

^10^Department of Ophthalmology, Faculty of Medicine, Health and Human Sciences, Macquarie University, New South Wales, Australia

^11^Department of Ophthalmology, Hadassah Medical Center, Faculty of Medicine, The Hebrew University of Jerusalem, Jerusalem, Israel

^12^Northern California Retina Vitreous Associates, Mountain View, California, USA

^13^Department of Ophthalmology, Institute of Vision Research, Gangnam Severance Hospital, Yonsei University College of Medicine, Seoul, Republic of Korea

^14^Casey Eye Institute at Oregon Health and Science University, Portland, OR, USA

^15^Department of Ophthalmology and Visual Sciences, The Chinese University of Hong Kong, Hong Kong Eye Hospital, People's Republic of China

^16^Department of Ophthalmology, Federal University of São Paulo, São Paulo, Brazil

^17^Department of Biomedical, Surgical and Dental Sciences, University of Milan, Milan, Italy

^18^Eye Clinic, IRCCS MultiMedica, Milan, Italy

^19^Harvard Medical School, Department of Ophthalmology, Massachusetts Eye and Ear, Boston, MA, USA

^20^Department of Retina and Vitreous, L V Prasad Eye Institute, Vijayawada, India

^21^Associação para a Investigação Biomédica e Inovação em Luz e Imagem, Coimbra, Portugal

^22^Faculdade de Medicina da Universidade de Coimbra, Coimbra, Portugal

^23^Macula and Retina Institute, Glendale and Los Angeles, CA, USA

^24^Molecular Insight Research Foundation, Glendale and Los Angeles, CA, USA

^25^University of California Davis, Davis, California, USA

^26^Mid Atlantic Retina, The Retina Service of Wills Eye Hospital, Philadelphia, PA, USA

^27^Asociados de Macula Vitreo y Retina de Costa Rica, San José, Costa Rica

^28^The Canberra Hospital, Garran, ACT, Australia

^29^Sydney Eye Hospital, Sydney, NSW, Australia

^30^Department of Experimental and Clinical Medicine, Eye Clinic, Polytechnic University of Marche, Ancona, Italy

^31^Department of Ophthalmology, Hôpital Cochin, Assistance Publique-Hôpitaux de Paris, Paris, France

^32^Department of Ophthalmology, Graduate School of Medicine, University of the Ryukyus, Okinawa, Japan

^33^Fundación Oftalmológica Nacional, Universidad del Rosario, Bogotá, Colombia

**Author Contributions**

Conceptualization: JC; Data curation and Formal analysis: NH, AZ, PG, BM, RN, GG, JEC, SS, LC, GP, PC, PS, PAS, LS, NJ, NI, CVT, HW, AC, LFD, FH, JW, YA, GP, CARF; Funding acquisition: JC; Investigation and Methodology: NKS, NH, JC; Project administration: JC; Resources: SG, ML, CCW, ER, GY, MKG, EB, MRM, RNK, ZK, MBP, SV, LAB, KS, RS, LHL, ATF, LW, JC; Software and Supervision: NKS, JC; Validation and Visualization: SG, ML, CCW, ER, GY, MKG, EB, MRM, RNK, TL, LP, SK, PR, ZK, MBP, SV, LAB, MK, NKS, KS, MZ, HK, FR, RS, LHL, ATF, LW, JC; Writing – original draft: NKS; Writing – review and editing: SG, ML, CCW, ER, GY, MKG, EB, MRM, RNK, TL, LP, SK, PR, ZK, MBP, SV, LAB, MK, NKS, KS, MZ, HK, FR, RS, LHL, ATF, LW, JC.

**Individual Author Details**

**Nasiq Hasan, MD^1,2^**

**Email:** nasiq.imtiaz@gmail.com

**Affiliation:** University of Pittsburgh Medical Center, Pittsburgh, PA, USA; University of Pittsburgh School of Medicine, Pittsburgh, PA, USA

**Highest Degree:** MD

**Arman Zarnegar, MD^1,2^**

**Email:** armanzarnegarr@gmail.com

**Affiliation:** University of Pittsburgh Medical Center, Pittsburgh, PA, USA; University of Pittsburgh School of Medicine, Pittsburgh, PA, USA

**Highest Degree:** MD

**Jay Chhablani, MD^1,2^**

**Email:** jay.chhablani@gmail.com

**Affiliation:** University of Pittsburgh Medical Center, Pittsburgh, PA, USA; University of Pittsburgh School of Medicine, Pittsburgh, PA, USA

**Highest Degree:** MD

**Priyanka Gandhi, MD^2^**

**Email:** phgandhi28@gmail.com

**Affiliation:** University of Pittsburgh School of Medicine, Pittsburgh, PA, USA

**Highest Degree:** MD

**Carmen Antía Rodríguez-Fernández, MS^3^**

**Email:** carmenantia@gmail.com

**Affiliation:** Department of Ophthalmology, Hospital Universitari de Bellvitge, L'Hospitalet de Llobregat, Catalunya, Spain

**Highest Degree:** MS

**Luis Arias Barquet, MD, PhD^3^**

**Email:** luisariasbarquet@gmail.com

**Affiliation:** Department of Ophthalmology, Hospital Universitari de Bellvitge, L'Hospitalet de Llobregat, Catalunya, Spain

**Highest Degree:** MD, PhD

**Jessica Cao, MD^4^**

**Email:** jessica.anning.cao@gmail.com

**Affiliation:** Retina Consultants of Texas, Retina Consultants of America, Blanton Eye Institute, Houston, TX, USA

**Highest Degree:** MD

**Stanley Saju, DO^4^**

**Email:** stanley.saju@gmail.com

**Affiliation:** Retina Consultants of Texas, Retina Consultants of America, Blanton Eye Institute, Houston, TX, USA

**Highest Degree:** DO

**Charles C. Wykoff, MD, PhD^4^**

**Email:** charleswykoff@gmail.com

**Affiliation:** Retina Consultants of Texas, Retina Consultants of America, Blanton Eye Institute, Houston, TX, USA

**Highest Degree:** MD, PhD

**Lisa Checchin, MD^5^**

**Email:** checchin.lisa@hsr.it

**Affiliation:** Ophthalmology Unit, IRCCS San Raffaele Scientific Institute, Milan, Italy

**Highest Degree:** MD

**Maurizio Battaglia Parodi, MD^5^**

**Email:** maubp@yahoo.it

**Affiliation:** Ophthalmology Unit, IRCCS San Raffaele Scientific Institute, Milan, Italy

**Highest Degree:** MD

**Gabriele Piccoli, MSc, OD^5^**

**Email:** gabriele.piccoli95@gmail.com

**Affiliation:** Ophthalmology Unit, IRCCS San Raffaele Scientific Institute, Milan, Italy

**Highest Degree:** MSc, OD

**Lorenzo Pili, MD^5^**

**Email:** pili.lorenzo@hsr.it

**Affiliation:** Ophthalmology Unit, IRCCS San Raffaele Scientific Institute, Milan, Italy

**Highest Degree:** MD

**Peranut Chotcomwongse, MD^6^**

**Email:** peranut.chot@gmail.com

**Affiliation:** Vitreoretina Unit, Department of Ophthalmology, Rajavithi Hospital, Rungsit University, Bangkok, Thailand

**Highest Degree:** MD

**Paisan Ruamviboonsuk, MD^6^**

**Email:** paisan.trs@gmail.com

**Affiliation:** Vitreoretina Unit, Department of Ophthalmology, Rajavithi Hospital, Rungsit University, Bangkok, Thailand

**Highest Degree:** MD

**Lorenzo Ferro Desideri, MD, PhD^7^**

**Email:** lorenzoferrodes@gmail.com

**Affiliation:** Department of Ophthalmology, Northwestern University Feinberg School of Medicine, Chicago, IL, USA

**Highest Degree:** MD, PhD

**Manjot K. Gill, MD, FASRS^7^**

**Email:** mgill@nm.org

**Affiliation:** Department of Ophthalmology, Northwestern University Feinberg School of Medicine, Chicago, IL, USA

**Highest Degree:** MD, FASRS

**Felicia Hertkorn, MD^7^**

**Email:** felicia.hertkorn@gmail.com

**Affiliation:** Department of Ophthalmology, Northwestern University Feinberg School of Medicine, Chicago, IL, USA

**Highest Degree:** MD

**Marion R. Munk, MD, PhD^7,8,9^**

**Email:** marion_munk@hotmail.com

**Affiliation:** Department of Ophthalmology, Northwestern University Feinberg School of Medicine, Chicago, IL, USA; Inselspital, University Hospital Bern, Bern, Switzerland; Gutblick, Pfäffikon, Switzerland

**Highest Degree:** MD, PhD

**Halit Winter, MD^7^**

**Email:** halit.winter@gmail.com

**Affiliation:** Department of Ophthalmology, Northwestern University Feinberg School of Medicine, Chicago, IL, USA

**Highest Degree:** MD

**Adrian T. Fung, MBBS, MMED, FRANZCO^10^**

**Email:** adrian.fung@sydney.edu.au

**Affiliation:** Department of Ophthalmology, Faculty of Medicine, Health and Human Sciences, Macquarie University, New South Wales, Australia

**Highest Degree:** MBBS, MMED, FRANZCO

**Samer Khateb, MD, PhD^11^**

**Email:** samerkhateb@gmail.com

**Affiliation:** Department of Ophthalmology, Hadassah Medical Center, Faculty of Medicine, The Hebrew University of Jerusalem, Jerusalem, Israel

**Highest Degree:** MD, PhD

**Rahul N. Khurana, MD^12^**

**Email:** rnkhurana@gmail.com

**Affiliation:** Northern California Retina Vitreous Associates, Mountain View, California, USA

**Highest Degree:** MD

**Jay Wang, MD^12^**

**Email:** jaywang22@gmail.com

**Affiliation:** Northern California Retina Vitreous Associates, Mountain View, California, USA

**Highest Degree:** MD

**Min Kim, MD, PhD, MBA, FASRS^13^**

**Email:** minkim76@gmail.com

**Affiliation:** Department of Ophthalmology, Institute of Vision Research, Gangnam Severance Hospital, Yonsei University College of Medicine, Seoul, Republic of Korea

**Highest Degree:** MD, PhD, MBA, FASRS

**Yusuf Ashfaq, BS^14^**

**Email:** ashfaqy@ohsu.edu

**Affiliation:** Casey Eye Institute at Oregon Health and Science University, Portland, OR, USA

**Highest Degree:** BS

**Zachary Kroeger, MD^14^**

**Email:** kroegerz@ohsu.edu

**Affiliation:** Casey Eye Institute at Oregon Health and Science University, Portland, OR, USA

**Highest Degree:** MD

**Timothy Lai, MD^15^**

**Email:** tyylai@cuhk.edu.hk

**Affiliation:** Department of Ophthalmology and Visual Sciences, The Chinese University of Hong Kong, Hong Kong Eye Hospital, People's Republic of China

**Highest Degree:** MD

**Luiz H. Lima, MD^16^**

**Email:** luizlima9@gmail.com

**Affiliation:** Department of Ophthalmology, Federal University of São Paulo, São Paulo, Brazil

**Highest Degree:** MD

**Stela Vujosevic, MD, PhD, FEBO^17,18^**

**Email:** stela.vujosevic@unimi.it

**Affiliation:** Department of Biomedical, Surgical and Dental Sciences, University of Milan, Milan, Italy; Eye Clinic, IRCCS MultiMedica, Milan, Italy

**Highest Degree:** MD, PhD, FEBO

**Elizabeth Rossin, MD, PhD^19^**

**Email:** Elizabeth_Rossin@meei.harvard.edu

**Affiliation:** Harvard Medical School, Department of Ophthalmology, Massachusetts Eye and Ear, Boston, MA, USA

**Highest Degree:** MD, PhD

**Priya Shah, BS^19^**

**Email:** priyash4h@outlook.com

**Affiliation:** Harvard Medical School, Department of Ophthalmology, Massachusetts Eye and Ear, Boston, MA, USA

**Highest Degree:** BS

**Panisa Singhanetr, MD^19^**

**Email:** panisa.singhanetr@gmail.com

**Affiliation:** Harvard Medical School, Department of Ophthalmology, Massachusetts Eye and Ear, Boston, MA, USA

**Highest Degree:** MD

**Lucia Sobrin, MD^19^**

**Email:** lucia_sobrin@meei.harvard.edu

**Affiliation:** Harvard Medical School, Department of Ophthalmology, Massachusetts Eye and Ear, Boston, MA, USA

**Highest Degree:** MD

**Niroj Kumar Sahoo, MD^20^**

**Email:** nirojsahoo71@gmail.com

**Affiliation:** Department of Retina and Vitreous, L V Prasad Eye Institute, Vijayawada, India

**Highest Degree:** MD

**Ninan Jacob, MD^20^**

**Email:** jnbrother1@gmail.com

**Affiliation:** Department of Retina and Vitreous, L V Prasad Eye Institute, Vijayawada, India

**Highest Degree:** MD

**Rufino Silva, MD^21,22^**

**Email:** rufino.silva@oftalmologia.co.pt

**Affiliation:** Associação para a Investigação Biomédica e Inovação em Luz e Imagem, Coimbra, Portugal; Faculdade de Medicina da Universidade de Coimbra, Coimbra, Portugal

**Highest Degree:** MD

**Kent Small, MD^23,24^**

**Email:** kentsmall@hotmail.com

**Affiliation:** Macula and Retina Institute, Glendale and Los Angeles, CA, USA; Molecular Insight Research Foundation, Glendale and Los Angeles, CA, USA

**Highest Degree:** MD

**Carol Villafuerte-Trisolini, MD^25^**

**Email:** civillafuertetrisol@ucdavis.edu

**Affiliation:** University of California Davis, Davis, California, USA

**Highest Degree:** MD

**Glenn Yiu, MD, PhD^25^**

**Email:** gyiu@ucdavis.edu

**Affiliation:** University of California Davis, Davis, California, USA

**Highest Degree:** MD, PhD

**Sunir Garg, MD^26^**

**Email:** sunirgarg@gmail.com

**Affiliation:** Mid Atlantic Retina, The Retina Service of Wills Eye Hospital, Philadelphia, PA, USA

**Highest Degree:** MD

**Bita Momenaei, MD^26^**

**Email:** momenaeib@gmail.com

**Affiliation:** Mid Atlantic Retina, The Retina Service of Wills Eye Hospital, Philadelphia, PA, USA

**Highest Degree:** MD

**Roselind Ni, MD^26^**

**Email:** niroselind@gmail.com

**Affiliation:** Mid Atlantic Retina, The Retina Service of Wills Eye Hospital, Philadelphia, PA, USA

**Highest Degree:** MD

**Lihteh Wu, MD^27^**

**Email:** lihteh@gmail.com

**Affiliation:** Asociados de Macula Vitreo y Retina de Costa Rica, San José, Costa Rica

**Highest Degree:** MD

**Michael Zhang, MD^28,29^**

**Email:** mgzhang90@gmail.com

**Affiliation:** The Canberra Hospital, Garran, ACT, Australia; Sydney Eye Hospital, Sydney, NSW, Australia

**Highest Degree:** MD

**Giulia Gregori, MD^30^**

**Email:** g.gregori98@gmail.com

**Affiliation:** Department of Experimental and Clinical Medicine, Eye Clinic, Polytechnic University of Marche, Ancona, Italy

**Highest Degree:** MD

**Marco Lupidi, MD^30^**

**Email:** dr.marco.lupidi@gmail.com

**Affiliation:** Department of Experimental and Clinical Medicine, Eye Clinic, Polytechnic University of Marche, Ancona, Italy

**Highest Degree:** MD

**Elodie Bousquet, MD^31^**

**Email:** bousquetelodie29@gmail.com

**Affiliation:** Department of Ophthalmology, Hôpital Cochin, Assistance Publique-Hôpitaux de Paris, Paris, France

**Highest Degree:** MD

**Andrea Coletto, MD^31^**

**Email:** coletto.andrea94@gmail.com

**Affiliation:** Department of Ophthalmology, Hôpital Cochin, Assistance Publique-Hôpitaux de Paris, Paris, France

**Highest Degree:** MD

**Naoya Imanaga, MD^32^**

**Email:** nimanaga23@gmail.com

**Affiliation:** Department of Ophthalmology, Graduate School of Medicine, University of the Ryukyus, Okinawa, Japan

**Highest Degree:** MD

**Hideki Koizumi, MD^32^**

**Email:** hkoizumi0303@gmail.com

**Affiliation:** Department of Ophthalmology, Graduate School of Medicine, University of the Ryukyus, Okinawa, Japan

**Highest Degree:** MD

**Francisco Rodriguez, MD^33^**

**Email:** fjrodriguez.retina@gmail.com

**Affiliation:** Fundación Oftalmológica Nacional, Universidad del Rosario, Bogotá, Colombia

**Highest Degree:** MD
